# Ethical issues in the development and implementation of nutrition-related public health policies and interventions: A scoping review

**DOI:** 10.1371/journal.pone.0186897

**Published:** 2017-10-26

**Authors:** Thierry Hurlimann, Juan Pablo Peña-Rosas, Abha Saxena, Gerardo Zamora, Béatrice Godard

**Affiliations:** 1 Public Health Research Institute of the University of Montreal (IRSPUM), Montreal, Canada; 2 Evidence and Programme Guidance, Department of Nutrition for Health and Development, World Health Organization, Geneva, Switzerland; 3 Department of Information, Evidence and Research, World Health Organization, Geneva, Switzerland; Pennsylvania State University College of Medicine, UNITED STATES

## Abstract

**Background:**

The limited integration of ethics in nutrition-related public health policies and interventions is one major concern for those who have the task of implementing them. Ethical challenges that are overlooked during the development of such interventions could raise serious ethical issues during their implementation and even after. As a result, these decision makers need technical support and ethical guidance for adaptation of interventions to local (cultural, social, economic, etc.) contexts.

**Aim:**

The goal of this scoping review is to delineate and “map” the range of ethical issues in nutrition-related public health interventions, as well as the range of the various fields in which they may arise.

**Methods:**

A scoping review of empirical research and conceptual literature was conducted following the framework of Arksey and O’Malley. Searches using PubMed with Medical Subject Headings (MeSH) categories and Advanced Search Builder as well as in the Global Health Library were performed. The final sample consists of 169 publications.

**Results:**

The ethics of public health prevention or treatment of obesity and non-communicable diseases is the most explicitly and frequently discussed subject. In comparison, ethical issues raised by public health interventions in the fields of undernutrition, breastfeeding, vitamin/mineral supplementation and food fortification, food security, food sustainability and food safety are addressed in a lower proportion of the sample. The results illustrate the various natures, types, and scopes of existing public health nutrition-related interventions, and the various ethical issues that may be raised by these interventions, in addition to the numerous and different contexts in which they may be implemented.

**Discussion:**

The ethical issues faced in the development and implementation of nutrition-related public health interventions are varied and cannot be equated with, nor generalized about, when dealing with specific activities in this field. More importantly, these ethical issues cannot be managed without a careful consideration for the complexity of contexts in which nutrition-related interventions are expected to be implemented. These interventions engage a variety of actors with diverse perspectives and interests. We discuss these challenges and also comment on the importance of considering ethical impacts in the monitoring and evaluation of such interventions.

**Conclusion:**

General ethical frameworks or recommendations–although useful–cannot be expected to provide policy makers, implementators and other public health personnel with sufficient practical ethical guidance as they cannot consider and anticipate the particularities of all specific nutrition-related public health interventions and the complexity of the contexts in which they are implemented. Further research is needed in order to develop more targeted ethical frameworks.

## Introduction

There is increasing support for developing frameworks for ethical considerations in the development and implementation of nutrition-related public health interventions [1–4]. Kass defines ethical frameworks as an analytic tool “designed to help public health professionals consider the ethics implications of proposed interventions, policy proposals, research initiatives, and programs” (p. 1777 in [5]). There have been systematic efforts to articulate such frameworks to guide public health interventions [5–11]. A number of general moral considerations have been addressed, that include: producing benefits, preventing harms, distributing health benefits fairly; respecting individual autonomy and liberty of action; respecting and fulfilling universal human rights; protecting vulnerable groups from marginalization and stigmatization; building and maintaining trust. Efforts have been made to produce frameworks of unranked principles [10] or theories of social justice [9], among other more practical approaches [5], but much work needs to be done to translate these general ethical considerations or some subset of them into guiding principles and frameworks for nutrition-related public health policy and intervention. Such work entails identifying actual and potential ethical issues, defining them, determining their scope, specifying criteria for resolving conflicts among them, and so on. The limited integration of ethics in nutrition-related public health policies and interventions [12] is one major concern for those who have the task of implementing policies, as the ethical challenges that were overlooked during the development of an intervention could raise serious ethical issues during its implementation and even after. As a result, these decision makers need technical support and ethical guidance for adaptation of interventions to local (cultural, social, economic, etc.) contexts. Depending on the flexibility that is given to them by the policy, it is expected that all implementation of a given intervention should be preceded by an examination of the basic ethical principles that serve as a justification for the particular ethical prescriptions and evaluations of human actions [13]. As an example, in 2002, the Public Health Leadership Society elaborated 12 principles of the ethical practice of public health [14].

The following scoping review is part of an assignment commissioned by the World Health Organization. The authors were asked to conduct a scoping review of the literature on ethical issues in nutrition-related public health activities for ethics-related guidance to be developed by the WHO Department of Nutrition for Health and Development. This review aims to ascertain and delineate the range of ethical issues in nutrition public health interventions that could serve as a basis to increase efforts to address the ethics of public health nutrition, and, in future steps, to further develop and integrate ethical frameworks in this field.

## Materials and methods

Scoping reviews are broad by nature are used to delineate, to map the key concepts underpinning a field of research as well as to clarify working definitions, and/or the conceptual boundaries of a topic that encompass a range of interventions and outcome measures (p. 6–8 in [15]). This scoping review of empirical research and conceptual literature follows the framework of Arksey and O’Malley [16], which involves: 1) defining a research question, 2) identifying and selecting relevant studies/publications, 3) charting resulting data, 4) interpreting, summarizing, and reporting the results.

### Step 1: Identifying the research question

The purpose of this review is to delineate and “map” the range of ethical issues in nutrition-related public health interventions, as well as the range of the various fields in which they may arise. It covers the ethical issues that may arise at all levels in nutrition-related interventions, including the development of policies, guidelines, recommendations, and interventions at the population level, as well in their implementation and evaluation [17–21].

### Step 2: Identifying relevant articles and selecting articles

**Fig 1:**
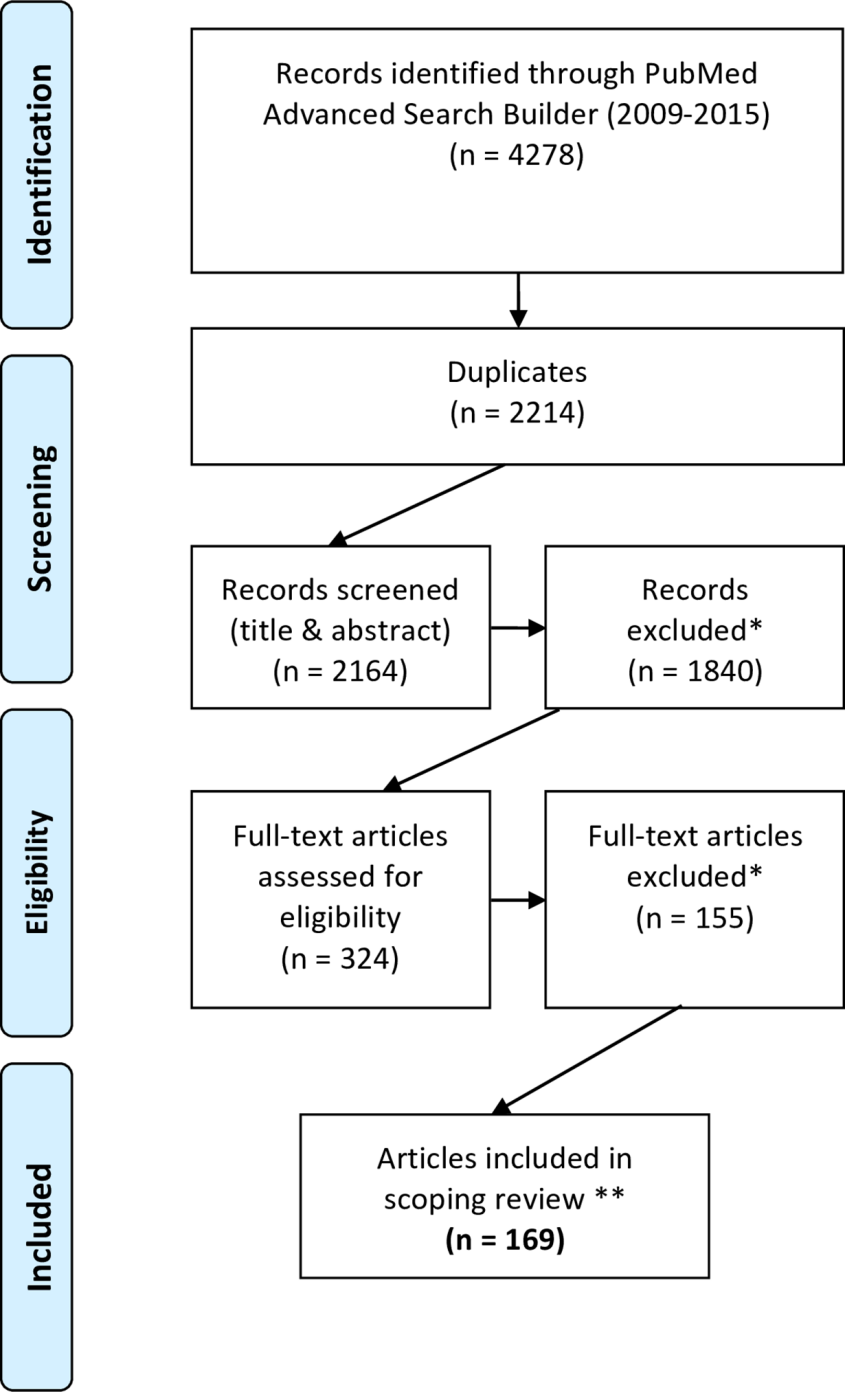
Search strategy. Adapted from Moher D, Liberati A, Tetzlaff J, Altman DG, The PRISMA Group. Preferred Reporting Items for Systematic Reviews and Meta-Analyses: The PRISMA Statement. PLoS Med. 2009;6(7): e1000097. doi:10.1371/journal.pmed1000097. *Exclusion criteria: Non-human studies; public health surveillance not related to nutrition, patents issues, articles that do not address ethical issues, publications focusing *only* on diets in patients with one specific disease—unless a significant number of papers addressing the same disease can be found; end of life and parenteral nutrition; nutrition and sport; basic and clinical nutrition research not related to public health interventions and policies; research ethics; education in ethics, bariatric surgery; articles focusing on physical activity only in the prevention or treatment of obesity; publications about hunger strikes; editorials, short news, article not in French or English. 11 publications were excluded after full-text screening, as it appears that their identification with the keyword “ethic*” in PubMed database only resulted from: a) the mention of “ethical” approval (or exemption of ethical approval) from a research ethics board or, b) the mention of the affiliation of the author (an institute/department of ethics) in the body of the text. These 11 articles were not addressing any ethical issue. However, articles that are clearly describing ethical issues (even without using the word “ethics” or “ethical”) were kept in our final sample even if their identification in PubMed only resulted from the mention of “ethical” approval, the title of the journal in which they were published (Journal of Medical Ethics and Law), or a classification by PubMed under the “ethics” category (n = 24). ** Final sample (S2 Table) contains research articles, reviews, feature articles and commentaries.

A time frame for publications was set in order to focus on recent developments (2009 and 2015). We performed a search with PubMed Advanced Search Builder using truncation and combinations of the following keywords: “ethics”, “health”, “public health”, “global health”, “nutrition”, “undernutrition”, “malnutrition”, “recommendation”, “guideline”, “activity”, “policy”, “intervention”, and “evidence” (see S1 Table). Fig 1 outlines the search strategy. After de-duplication, we screened the records to assess eligibility and analyse the full-text manuscripts. The final sample consists of 169 publications (S2 Table).

It is difficult to determine to what extent issues linked to ethics have to be dealt with in an article before it can be considered as actually addressing “ethical issues”. For instance, articles that briefly mention the importance of cultural factors in the implementation of nutrition policies or the need to involve target populations in the development of these policies are, in effect, addressing “ethical issues”, yet without explicitly mentioning it, and thus, may not have been identified as such using PubMed Advanced Search Builder. Similarly, key principles in bioethics can be associated with many dimensions of a public health policy, and authors considering these issues may do so without explicitly identifying them as “ethical” issues. Thus, in order to ensure that we sufficiently captured the field of ethics, we also conducted a search on Pubmed using Medical Subject Headings (MeSH) categories and the Global Health Library–which includes Latin American and Caribbean Health Sciences Literature (LILACS), the World Health Organization library database (WHOLIS), the African Index Medicus (AIM), the Western Pacific Region Index Medicus (WPRIM), and the Index Medicus for the Eastern Mediterranean Region (IMEMR). We used combinations of the following subject headings: “Morals”, “Nutrition disorders”, “Nutrition Policy and Nutrition Sciences”. In the PubMed MeSH system, “Ethics” is a category that is included in “Morals” (Time limit: 2009–2015). This latter category encompasses articles that were classified under a number of other subcategories, such as “Principle-Based Ethics”, and, thus, includes terms such as “beneficence”, “autonomy” and “justice”. Consequently, the results of our search with MeSH should theoretically include all articles addressing these principles.

After screening, the final sample resulting from this MeSH search (n = 157) included 34 articles that were already included in the first sample of this scoping review. A vast majority of the articles identified through the MeSH search focused on obesity (over 71%) and the relevance of many articles was doubtful with regard to the purpose of this scoping review (for instance, articles that focused on philosophical and moral theories are only tenuously related to concrete actions and practical ethical issues in public health nutrition-related interventions). In every case, we observed that all the fields and subjects addressed by the publications identified through the Pubmed search with MeSH categories, as well as in the Global Health Library, were covered by the initial sample obtained using PubMed’s Advanced Search Builder. Therefore, this scoping review focuses solely on that first sample.

### Step 3: Charting resulting data

**Fig 2:**
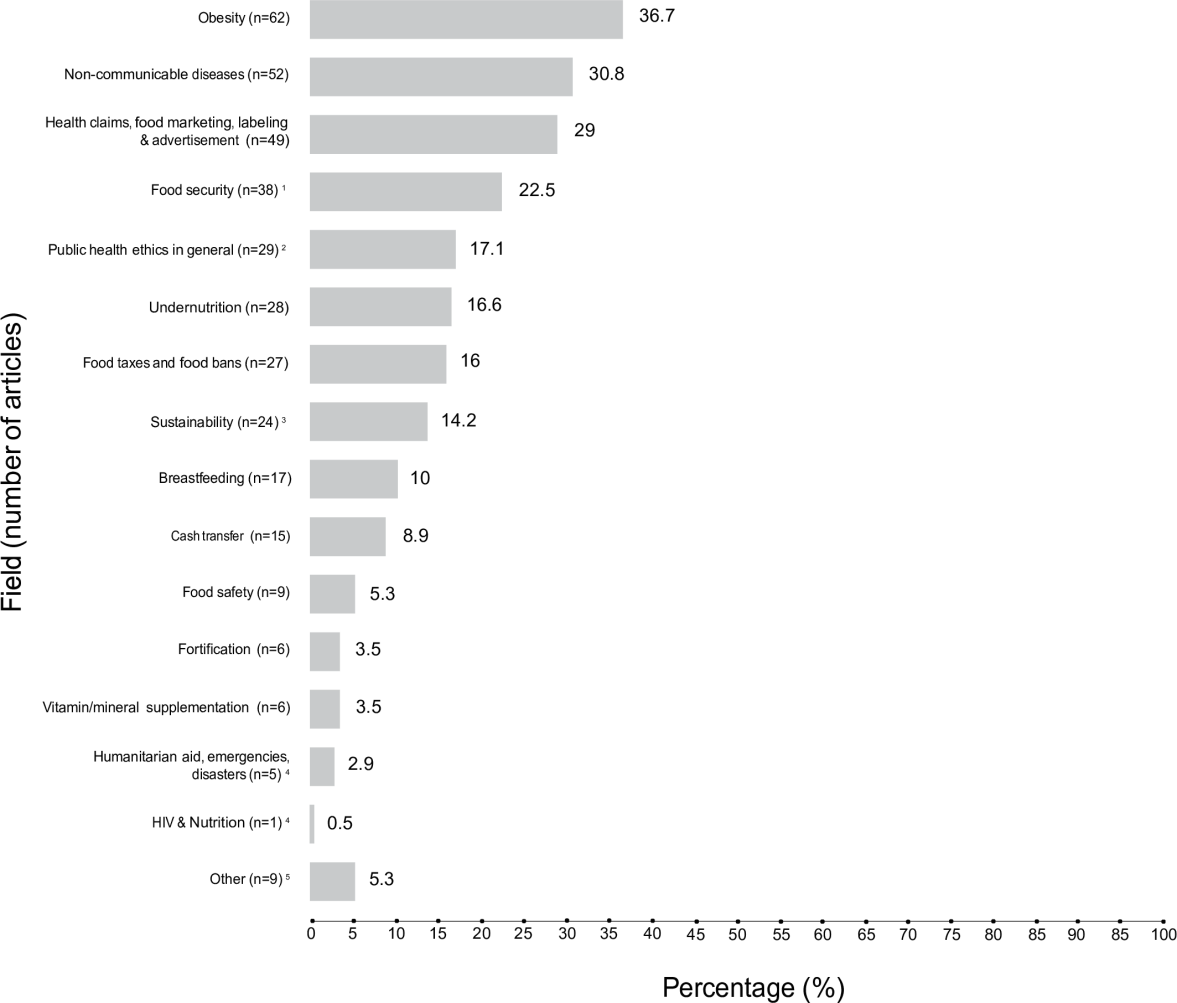
Main fields of public health and nutrition addressed in the whole sample (n = 169). Total of articles: 169. A same article may appear in more than one category. Note that 8 articles could not be accessed online through the University of Montreal electronic resources for full content analysis. Thus, these articles might have appeared in additional categories that could not be identified only in abstract and title. ^1^Includes food access disparities; ^2^This category encompasses all articles that address ethics in public health in general (e.g., ethical framework in public decision-making and that do not focus on nutrition-related interventions); ^3^Including in food production; ^4^These articles [12, 22–26] are cited in this paper when relevant (most of them are classified in another field), but given their small number, there is no specific section about HIV and nutrition and nutrition-intervention in humanitarian aid in this paper; ^5^Articles that could not be classified in one (at least) of the other fields. Several of these articles address conflict of interests, sponsorships and partnerships in public health and nutrition [27–30]^.^ The other articles focus on behaviors, perceptions and food choice motives [31–33], nutritionism and the ethics of the commercialization of food [34], and the implications for public health of appropriate information to consumers and health claims linked to polyphenols [35].These articles are not discussed further in this paper, but are included in the statistics.

**Fig 3:**
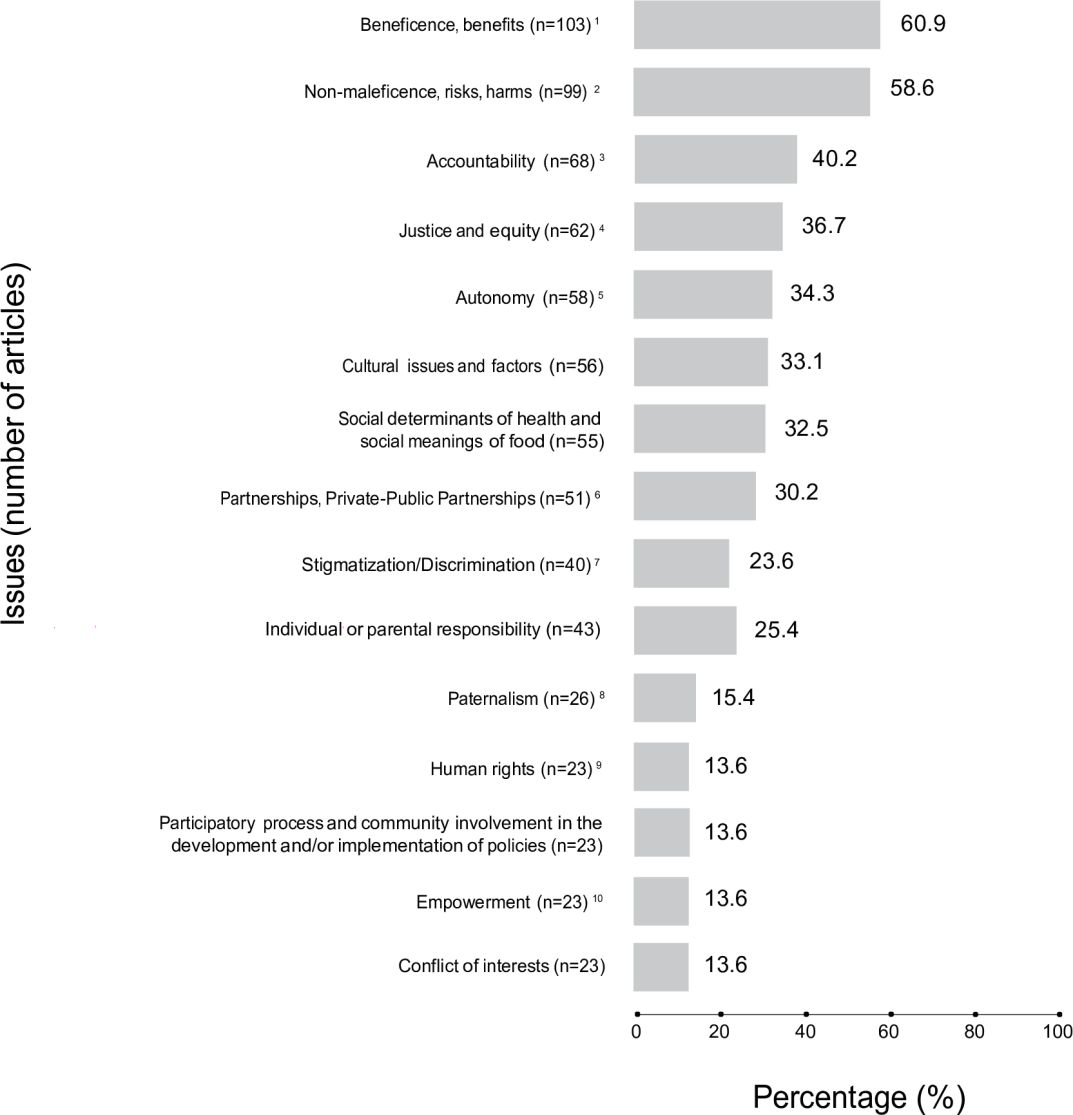
Most addressed issues linked to ethics in the whole sample (n = 169). Articles may appear in more than one category, and certain categories are directly linked to others (e.g., paternalism and empowerment can be linked to autonomy; as can stigmatization to justice). Note that 8 articles could not be accessed online through the University of Montreal electronic resources for full content analysis. Thus, these articles might have appeared in additional categories that could not be identified only through abstract and title. It is worth pointing out that searches within articles with specific words has limitations, as papers can actually address similar issues without using these specific terms. ^1^Most articles address benefits of interventions to a certain extent, often without explicitly considering beneficence as an ethical requirement. Search was limited to the explicit use of the word “benefit” in the articles, except for the articles appearing in the category “Empowerment”, which were all included here. ^2^Search was limited to the explicit use of the words “risk”, “harm” and “non-maleficence”, except for the articles appearing in category “Paternalism” and “Stigmatization-Discrimination”, which were all included here. Articles may address risks of interventions to a certain extent without explicitly associating them with an ethical principle. ^3^Accountability is understood as state, institutions, and organizations’ responsibility in their activities. Search was limited to the explicit use of the word “accountab*”. ^4^Search was limited to the explicit use of the words “justice” and “equity”. ^5^Search within articles with the words “freedom”, “choice” and “autonomy”. ^6^This category includes all articles that describe and comment on collaborations/partnerships between organizations, corporations, institutions, both in the public and private sectors. ^7^Search was limited to the explicit use of the word “stigma*” and “discrimin*” in the articles. ^8^Search was limited to the explicit use of the word “paternalis*” in the articles. ^9^Search was limited to the explicit use of the words “human rights” in the articles. ^10^Search was limited to the explicit use of the word “empower*” in the articles.

The categorization of articles into main fields of public health nutrition was a first step in charting the data (Fig 2). The next stage involved additional ‘charting’ of key items, particularly in the field of ethics (Fig 3). In this field, the principles of beneficence, non-maleficence, autonomy and justice are the most commonly used. These principles are broad concepts that may be applied to more specific ethical issues, depending on the circumstances and contexts in which these issues may arise or on the perspectives from which there are analysed. Beneficence includes considerations of the cost-effectiveness and utility of interventions, as well as for the social impact of those interventions on populations and individuals. Similarly, non-maleficence encompasses considerations about potential physical harms, as well as social risks. An ethical issue such as empowerment, for instance, can be linked to both beneficence and autonomy. Stigmatization, as another example, has ethical dimensions that are related to non-maleficence as well as to the principle of justice (see legend of Fig 3).

Given the different nature of risks and benefits of public health interventions, we identified specific, recurring issues linked to ethics during the data extraction process (see Fig 3). All publications were screened a second time to determine whether (or not) these issues were being addressed. We extracted the following data from each record: author(s), year of publication, type of publication, aims of study or subject of article, outcomes/conclusion, target populations, summary of ethical aspects addressed, and keywords.

### Step 4: Collating, summarizing and reporting results

Table 1 summarizes the frequencies at which specific ethical issues are discussed in each public health nutrition field. The ethical issues linked to challenges in implementation and evaluation of public health nutrition policies and intervention are presented in Tables 2 and 3. Results for each field of public health nutrition are presented, developed and commented in the following sub-sections.

**Table 1:** Most addressed issues linked to ethics in the whole sample (n = 169), % per field.

	Public health ethics (no focus on nutrition)	Obesity	Non- communicable diseases	Food security	Under-nutrition	Sustainability	Breastfeeding	Food safety	Food fortification	Vitamin / mineral supplement
Number of articles	(n = 29)	(n = 62)	(n = 52)	(n = 38)	(n = 28)	(n = 24)	(n = 17)	(n = 9)*	(n = 6)*	(n = 6)*
**Justice and equity**	86.2	37.1	28.8	52.6	46.4	54.2	23.5	33.3	33.3	16.6
**Beneficence, benefits**	75.9	58.1	75	55.3	39.3	54.2	58.8	55.5	50	33.3
**Non-maleficence, harms, risks**	75.9	62.9	63.5	50	35.7	50	52.9	44.4	50	33.3
**Accountability**	65.5	46.8	30.8	44.7	50	58.3	29.4	44.4	50	16.6
**Individual/parental autonomy**	48.3	43.5	34.6	28.9	21.4	16.7	41.2	11.1	33.3	-
**Stigmatization / discrimination**	41.4	40.3	13.5	21	10.7	12.5	11.8	-	-	-
Partnerships–PPPs**	41.4	40.3	25	55.3	46.4	62.5	41.2	33.3	33.3	16.6
**Community involvement–Participatory process**	31	12.9	7.7	28.9	21.4	29.2	11.8	22.2	-	-
**Social determinants of health**	37.9	48.4	38.8	63.1	50	50	23.5	22.2	16.6	-
**Individual or parental responsibility**	27.6	46.8	21.1	31.6	17.8	20.8	23.5	-	16.6	16.6
**Cultural factors and issues**	27.6	37.1	40.4	50	39.3	58.3	41.2	33.3	16.6	16.6
**Empowerment**	24.1	11.3	19.2	15.8	7.1	8.3	11.8	11.1	-	-
**Paternalism**	24.1	22.6	11.5	7.9	3.6	4.2	23.5	-	33.3	-
**Human rights**	20.7	16.1	15.4	34.2	35.7	29.2	11.8	22.2	16.6	33.3
**Conflict of interests**	13.8	16.1	15.4	5.3	10.7	16.7	23.5	22.2	33.3	16.6
**Stakeholders’ perceptions**	6.9	9.7	15.4	5.3	10.7	8.3	23.5	-	33.3	16.6

**Table 2:** Number of articles mentioning or addressing challenges linked to ethical issues in the implementation of public health policies or interventions, per field (n = 55).

Fields	Number of articles	References
**Obesity**	26	[1, 2, 43, 57, 67, 70–73, 77, 79–84, 88, 89, 96, 98–100, 103, 106, 111, 112]
**Food security**	15	[26, 57, 71, 77, 79, 96, 98–100, 155, 160, 162, 167, 178, 181]
**Breastfeeding**	12	[38, 51, 67, 72, 73, 96, 162, 164, 165, 169, 171, 172]
**Sustainability**	12	[26, 48, 57, 71, 96, 99, 100, 116, 155, 167, 178, 182]
**Public health ethics**	12	[12, 38, 41–43, 46–48, 51, 54, 57, 58]
**Noncommunicable diseases**	9	[70, 79, 80, 88, 96, 98, 116, 126, 130]
**Undernutrition**	9	[96, 98, 151, 155, 160, 162, 164, 165, 167]
**Food fortification**	4	[96, 164, 167, 175]
**Vitamin/mineral supplementation**	2	[67, 151]
**Food safety**	2	[27, 178]
Others*	2	[12, 26]

Note that one same article may appear in more than one field.

* Includes the categories “HIV and nutrition”, “Humanitarian aid” and “Others” categories (see Fig 2).

**Table 3:** Number of articles addressing challenges linked to ethical issues in the evaluation of public health policies/interventions and to evidence, per field (n = 62)*.

Fields	Number of articles	References
**Obesity**	27	[1–4, 43, 56, 57, 61, 62, 71–75, 77, 78, 84, 92, 93, 96, 97, 99, 105, 106, 109, 112, 114]
**Public health ethics**	15	[12, 38, 39, 41–43, 46–48, 51, 54, 56–58, 61]
**Food security**	15	[26, 56, 57, 71, 77, 96, 99, 109, 143, 152, 155, 157, 160, 178, 180]
**Sustainability**	15	[26, 48, 56, 57, 71, 96, 99, 109, 116, 143, 155, 157, 178, 180, 182]
**Noncommunicable diseases**	14	[56, 74, 75, 78, 96, 109, 116, 127, 131, 139–141, 144, 146]
**Undernutrition**	12	[24, 25, 56, 96, 109, 143, 151, 152, 155, 157, 160, 164]
**Breastfeeding**	8	[38, 51, 72, 73, 96, 152, 164, 171]
**Food safety**	5	[27, 109, 143, 178, 180]
**Food fortification**	3	[61, 96, 164]
**Vitamin/mineral supplementation**	2	[74, 151]
Others**	5	[12, 24–26, 185]

Note that one same article may appear in more than one field.

*Were excluded 8 articles focusing on nutrigenomics/nutrigenetics and addressing issues about evidence of nutrigenomics/nutrigenetics tests validity and utility (and not evidence/evaluation of public health interventions as such) See section “Ethical issues in prevention and treatment of noncommunicable diseases through dietary interventions”.

** Includes the categories “HIV and nutrition”, “Humanitarian aid” and “Others” categories (see Fig 2).

## Results

### Ethical issues in public health policies

Twenty-nine articles (17,1% of the whole sample) explicitly address ethics in public health [12, 22, 23, 36–61] (see Table 1). While some of these articles do not focus on nutrition-related interventions in particular, they were included in our sample as they provide a useful picture of the background of this scoping review as well as describe current challenges in the field of ethics and public health that apply to various types of public health interventions. Justice and equity are addressed in a vast majority of these articles, as they constitute one of the core principles in public health ethics and illustrate the strong link that exists between population health and social inequities. Several of these articles stress the lack of integration of ethics and ethical guidance in public health policies and interventions [40, 43, 44, 48, 50, 55, 57, 61]. Education and/or the assistance in ethics while developing/implementing public health interventions is also recommended [12, 40, 44, 53, 55]. Ten articles comment on existing and/or propose frameworks for an ethical evaluation of public health interventions in various contexts (including in health promotion, allocation decisions in humanitarian aid, health governance, and the monitoring and evaluation of interventions) [12, 23, 36, 39, 46, 48, 54, 57, 58, 61].

### Ethical issues in obesity prevention and treatment

Although there are broader actions that aim at promoting healthy diets in general, the ethics of public health prevention or treatment of obesity is the most explicitly and frequently discussed subject: 62 articles (36,7%) address this topic (see Table 1) [1–4, 43, 56, 57, 60–114]. There is a significant variety in the nature, scope, population targets and types of public health interventions in this field.

Several authors address ethical issues linked to regulations on and/or control of health claims & nutrition, as well as of food marketing, advertisement and labelling [57, 60, 61, 64, 67, 70, 78, 82, 88, 91–93, 95–99, 103–109, 113, 114], for instance the ethical acceptability of food marketing to children, management of competing commercial and public health interests, and the need to improve labelling so that to promote individual responsibility. Ethical issues raised by food bans and food taxes interventions–in particular sugar taxes–are mainly linked to the acceptability of restricting consumer choice, and to the risks that such interventions exacerbate socioeconomic inequalities [3, 4, 65, 79, 82–85, 87, 93, 95, 98–100, 102, 106, 108]. Public health interventions aimed at promoting access to healthy foods through incentives such as food stamps and cash transfers are considered by several authors as ethically problematic, in particular in terms of excessive paternalism, infringement of individual autonomy and risks of stigmatization [4, 57, 68, 79, 95, 97, 99, 100, 106, 109, 113].

Much emphasis is made on the unintended effects of obesity prevention programs, such as stigmatization and discrimination. Several papers describe the barriers that impede personal empowerment and respect for individual autonomy in the choice of food and lifestyle. The necessity to consider social determinants of obesity and cultural factors is also stressed in many of these articles, along with the need to address underlying social inequities and the risk of increasing existing health disparities when implementing obesity prevention policies. The balance between individual and collective responsibility (including in terms of health authorities’ as well as food industries’ accountability) is described as an important ethical issue in 29 articles. Some authors provide ethical guidance by developing or commenting on ethical frameworks to guide the development and implementation of policies in the field of obesity prevention specifically [1–4, 57, 61, 65, 79, 84, 85, 89, 92, 96–99, 110].

### Ethical issues in prevention and treatment of noncommunicable diseases through dietary interventions

Most discussed medical conditions in the 52 articles (30,8%) [49, 56, 64, 70, 74, 75, 78–80, 87, 88, 94, 96, 98, 104, 109, 115–150] that focus on nutrition and the prevention or treatment of noncommunicable diseases are cardio-vascular diseases and diabetes (see Table 1). Twenty-one articles focus on nutrigenomics/nutrigenetics and/or epigenomics/epigenetics [49, 64, 74, 94, 118–123, 125, 126, 128, 129, 142, 143, 145, 147–150] and personalized nutrition. Note that three of these articles address or mention obesity from this angle and were also included in the section *Ethical issues in obesity prevention and treatment* [64, 74, 94]. While we are still far from concrete implementation of nutrigenomics/nutrigenetics or epigenomics/epigenetics in public health, authors have started to address the ethical issues that might be raised by their applications, such as the threat to individual autonomy, the excessive burden on personal responsibility, and the stigmatization of individuals who would not comply with personal dietary recommendations. Interventions relating to health claims and nutrition, as well as food marketing, advertisement and labelling and their role in the prevention of noncommunicable diseases are mentioned or addressed in 16 articles [70, 78, 88, 96, 98, 104, 109, 127, 131, 133, 135–139, 146]. Ethical issues raised by food bans–in particular transfat bans–and food taxes interventions, such as described in the previous section, are also addressed in the field of noncommunicable diseases [79, 87, 98, 131–140]. As shown in Table 1, considerations about cultural factors and social determinants of health are often discussed in this field, as well as health authorities’ and food industries’ accountability.

### Ethical issues in prevention and treatment of undernutrition

Prevention of undernutrition in public health appears as a field that has been under less ethical scrutiny compared to obesity and non-communicable diseases: 28 articles (16%) address this topic [24, 25, 56, 67, 90, 96, 98, 104, 109, 110, 143, 151–167] (see Table 1). The contexts in which ethical issues are addressed in the field of undernutrition are various and cannot be easily categorized. They include among others: prevention of acute malnutrition and cash interventions [24], food security for various targeted populations worldwide [98, 152, 156, 162, 163, 166], food stamp programs [68], inequalities between boys and girls in India [153], collaborations and participatory approaches in a specific program in Sub-Saharan Africa [157], guidance on maternal nutrition [164], food fortification [96,164], nutritional care of the elderly [154, 158], and innovations in capture fisheries for nutrition security [155]. Interventions relating to health claims and nutrition, food marketing, advertisement and labelling are mentioned or addressed in several articles, for instance in regard with breastmilk substitutes [96, 164–166].

A majority of these articles is organized around or address food security (see section *Ethical issues in food security* below). Consideration for social determinants of health, states and other stakeholders’ accountability, the challenges in partnerships (such as commercial versus public health interests) and the importance of community involvement as well as cultural factors for sustainability, and the issues of justice and equity in particular fair access to food are some of the most discussed issues (see Table 1).

### Ethical issues in breastfeeding practices

Breastfeeding is addressed in 17 articles (10%) [38, 51, 67, 72, 73, 96, 152, 153, 162, 164–166, 168–172] (see Table 1). Beyond the benefits and risks of public health interventions linked to breastfeeding, respect for mothers’ autonomy and their cultural values [168] in the promotion of breastfeeding, infants’ best interests [170], as well as the challenges that are raised by public-private partnerships in this field (e.g., conflicts of interest) are among the major ethical issues discussed in these articles. Several articles mention/address concerns relating to health claims, as well as marketing, advertisement and labelling of breastmilk substitutes, in particular concerns about aggressive marketing of infant formula by private companies that interferes with public health promotion of breastfeeding [67, 96, 164–166].

It is worth noting that Fetherstone and Leach [171] address public health policies promoting breastfeeding: they refer to the Nuffield Council of Bioethics ethical framework and includes the principles of utility, evidence base and effectiveness of action, fairness, accountability, costs and burdens, and community acceptance.

### Ethical issues in vitamin/mineral supplementation and food fortification

Six articles address vitamin/mineral supplementation [67, 74, 104, 151, 173, 174] and six comment on food fortification [61, 96, 164, 166, 167, 175] (see Table 1). No article *specifically* focuses on the ethical issues that could be raised by vitamin/mineral supplementation. The contexts in which vitamin/mineral supplementation is addressed are various; for instance: needs for additional skills and knowledge of pharmacists to support appropriate nutritional advice to consumers in pharmacy settings and marketing practices [67]; lack of clinical access to specific innovative nutrition/vitamin products and regulations about food labelling and health claims [173]; lack of education in populations about the use of vitamin supplements [174]; limitations of observational evidence in vitamin supplementation [74]; and risks associated with the combination of public health interventions of different natures, such as vaccines and vitamin A supplementation [151]. Similarly, food (or water) fortification is not the subject of extensive ethical analysis, except in regard to states and other stakeholders’ accountability and to the benefits of such interventions (see Table 1). Fortification is mentioned in the following contexts, for instance: ethical acceptability, benefits and challenges of artificial water fluoridation [61, 175]; challenges in the implementation of interventions in the field of maternal nutrition, including food fortification policies [164]; balance of benefits and risks of crop biofortification in the prevention of hunger [167]; and food fortification programmes and challenges raised by public-private partnerships, including by marketing communications [96].

### Ethical issues in food security

Food security is explicitly addressed in 38 articles (22,5%) [26, 56, 57, 68, 71, 77, 79, 86, 87, 90, 96, 98–101, 104, 109, 110, 124, 134, 143, 152, 153, 155, 157, 159–163, 166, 167, 176–181] (see Table 1). The concept of food security has various definitions and dimensions (food appropriateness, availability, accessibility, affordability, utilization and stability of supply) and ethical ramification [68, 79, 90, 104, 109, 143, 159–162, 167]. Food security (implying a fair access to food) is directly associated with the ethical principle of justice, and as such, is included in several proposed ethical frameworks [79, 98, 166]. Most authors stress that food security cannot be reached without due considerations for social determinants of health and cultural factors. In addition, incentives such as food stamps and cash transfers are rising ethical issues that were shortly described above [57, 68, 79, 99, 100, 109, 163]. Marketing and private-public partnerships may also impact food security and raise risks such as conflicts between commercial and public health interests [96]. Finally, availability of food may also depend on efficient food production, which can generate concerns about animal welfare [176, 177]. Other issues listed in Table 1 are also commonly covered.

### Ethical issues in food sustainability

There is a direct link between sustainability and food security (see previous section). Among the 24 papers (14,2%–see Table 1) addressing sustainability [26, 48, 56, 57, 71, 87, 96, 99, 100, 104, 109, 110, 116, 143, 155, 157, 166, 167, 176–178, 180, 182, 183], 19 also appear in the field of food security. The sustainability of nutrition-related public health interventions, as well as the sustainability of effective food production depend on numerous factors, among which ethical consideration for the importance of partnerships with local communities, of the accountability of governments and private firms, of cultural factors, of justice and equity (for instance fair trading), and social determinants of health (see Table 1). Sustainability is an ethical consideration included in the ethical framework for monitoring and evaluating public health interventions proposed by Gopichandran et al. [48], as well as in the ethical principles proposed by Singh et al. [166]. The concept is also addressed in the ethical frameworks reviewed by Ten Have et al. [57].

### Ethical issues in food safety

As shown in Table 1, nine articles (5,3%) address issues that are linked to food safety, notably: challenges for food safety at all stages of production, including considerations for cultural factors, health claims and labelling [64, 109, 143, 183]; conflicts of interests with food industry and in expert panels that advise government agencies and public health officials formulating nutrition and food safety policy [27, 184]; and ethical issues that are specific to genetically modified animals and crops, including in labelling (e.g., respect for the freedom of choice of consumers) and animal welfare [178–180].

### Ethical issues raised by challenges in implementation and evaluation of public health nutrition policies and interventions

Challenges in implementation of public health policies are mentioned or addressed in 55 articles (32,5%–see Table 2). While such challenges are not necessarily discussed in terms of “ethical issues”, they may have an impact on the ethics of nutrition-related interventions. Most challenges are associated with the complexity of contexts in which nutrition policies are expected to be implemented (in particular, how to consider specific local settings, as well as perspectives and interests of various actors). The ethical frameworks reviewed by some authors do not make a clear distinction between the development of policies and their implementation [38, 57, 84, 130]. Brown and Allison suggest six recommendations when considering the implementation of an obesity-targeted public health policy and assume that implementators can “[e]valuate whether the proposed policy addresses an exposure that can truly be considered a public health concern” (p. 343 in [84]). Similarly, Thomson et al. suggest a checklist to help prevent premature or inappropriate implementation of certain public health interventions and stress that “[a]nticipation of positive and negative unintended consequences should be integral to the planning, design and implementation of interventions that include incentives, helping to ensure that any benefits are maximised” (p. 19 in [38]).

In 62 articles (excluding 8 articles focusing on nutrigenomics/nutrigenetics and epigenomics/epigenetics), issues relating to the evaluation of public health interventions and/or to the evidence on which they are or should be grounded are explicitly addressed (see Table 3). While most of these publications do not focus explicitly on the ethical dimensions of these matters, they address the benefits (n = 41, i.e., 66,1%) and risks (n = 39, i.e. 62,9%) of nutrition-related interventions or policies. In terms of ethics, assessing evidence and evaluating effectiveness are crucial steps in the development and implementation of such interventions and policies. Interventions “should be implemented only in the face of a clear public health need and good data demonstrating effectiveness” (p. 4 in [57]).

The major challenges described in these articles are to determine what constitutes sound “evidence” in public health programs and the lack of data [61, 71, 74]. Moreover, most authors stress that scientific evidence alone (in particular analyses limited to measuring effects on health and/or cost-effectiveness) cannot guide and determine health policy and decisions for intervention [1, 2, 43, 45, 51, 61, 84, 114, 124]. Ethical aspects, including unexpected consequences–such as stigma, negative impact on autonomy and individual choice, and negative perceptions [38, 47, 92]–must be considered in the monitoring and evaluation of interventions [92]. Several authors stress the importance of including the public or targeted communities (including health workers involved in the intervention) in the evaluation process [38, 41, 51, 56]. These results are further discussed below.

## Discussion

The results of this scoping review show that nutrition-related public health interventions can take many forms and their nature, goals, scope, and population targets may vary considerably. Such interventions may also occur at different levels, in different contexts, with the collaboration of various stakeholders. As a result, the ethical issues faced in the development and implementation of nutrition-related public health interventions are varied and cannot be equated with, nor generalized about, when dealing with specific activities in this field. More importantly, these ethical issues cannot be managed without a careful consideration for the complexity of contexts in which nutrition-related interventions are expected to be implemented. These contexts engage a variety of actors with diverse perspectives and interests, who can influence the implementation of policies [67]. There can be multiple interpretations of a policy and the intentions of policy-makers do not necessarily determine how a policy will be interpreted [85]. In some circumstances, “implementators”, as well as other health professionals, communities, institutions, etc. involved in public health interventions, can be at odds with the content of a policy, given specific local contexts, community values or personal beliefs or practices [41, 51, 111]. For such reasons, many articles stress the importance of partnerships and community’s/stakeholders’ involvement in the development and the implementation of policies [26, 42, 160, 178]. Participatory processes and consultations could allow them to anticipate ethical issues such as implementation of policies that conflict with personal or community values [99]–or avoid them altogether. Likewise when confronted by policies that are not adapted to the local contexts, as cultural factors are particularly important when it comes to food [26, 111]. Partnerships and cross-sectoral collaborations may be needed at different levels, across different sectors, public or private [54, 165]. Some authors from our sample stress the importance of not working in silos [12, 85], one policy aimed at improving access to healthy food, for instance, may not be effective without the concurrent implementation of other social measures to reduce poverty or environmental barriers. The implementation of a policy may require additional measures that were not initially described or planned in the policy, so it is also possible that several policies must be implemented at the same time to be effective [71, 79]. Those in charge of implementing nutrition-related policies may also have to cope with significant political resistance [88], lobbying pressures [80, 88], bureaucracy [47], and the risks raised by the presence of potential conflicts of interests when developing partnerships [54, 96].

Those involved in implementation need training and/or support, technical assistance, resources and ethical guidance for the adaptation of interventions to (cultural, social, economic, political, etc.) local contexts [55, 73]. Ten Have et al. [57], for instance, assume that implementators keep a certain margin of choice in the implementation of policies. Yet, this will actually depend on the flexibility of the policy, and no author in our sample discusses to what extent those who are involved in its implementation can question the ethics concerning a policy or an intervention or what latitude is left to them in specific and local contexts. In any case, adaptation of policies to local settings and community values calls for an ethical review. In this respect, Behrmann’s ethical principles outlined as a “Guide in Implementing Policies for the Management of Food Allergies in Schools” constitute a solid example of an ethical framework that is sensitive to the context in which it must apply and to the specifity of the nutrition-related interventions that it covers [130]. As such, it is a relevant example that can be used to develop and adapt frameworks for other nutrition-related public health interventions.

Finally, some authors consider that any implementation of an intervention should be accompanied with a plan to monitor and evaluate its impact [12, 48], including its ethical impact [38]. What constitutes sound “evidence” in public health programs and interventions remains a controversial issue. Two types of evidence are relevant in this field: evidence about causes of ill health, and evidence about the efficacy and effectiveness of interventions [61]. While focus is mainly on evidence-based practices [3, 72–74, 99, 157, 182], such evidence is often hard to obtain [152], lacking or incomplete [3, 74, 109, 116]. One current challenge to building an evidence base is the lack of data and commonly used tools and indicators to measure the effectiveness (including the cost effectiveness) of initiatives and programs [42, 71, 74]. Moreover, evidence about the impact of an intervention on nutrition in one context may not necessarily apply to other contexts [24]. It is often suggested that in the face of significant public health issues, “doing something is better than doing nothing” (p. 342 in [43]) and “how could it hurt to try?” (p. 342 in [84]). This viewpoint implies that interventions could be implemented if they are *expected* to be effective, without waiting for sufficient evidence [1, 43]. Yet, this stance is obviously problematic from an ethical standpoint, as such interventions could have no beneficial outcomes or could even be unsafe [151]. Conversely, a lack of evidence and/or absence of data such as relevant indicators allowing the measure of a public health problem [116] can prevent the implementation of *needed* public health interventions, something stressed by Shrimpton [164] in his discussion of maternal nutrition and iron fortification in the prevention of anaemia. Such issues are linked to debate and controversies surrounding the application of the precautionary principle (e.g., in [186, 187]), including to public health actions [188]. It calls for a careful assessment of risks and benefits of any intervention before implementation, even if there is not available evidence of either.

While monitoring and evaluation are essential parts of any public health policy or program [48], and the generation of evidence is integral to the work of public health and health service providers [24, 39, 112], too many interventions have still not been subjected to careful evaluation to assess their impact [106, 112, 114, 151, 152]. In this context, policy-makers and “implementators” can be left in a void, without guidance to determine what constitutes sound evidence to justify an intervention, and what factors must be considered within such an evaluation. Interventions “should be implemented only in the face of a clear public health need and good data demonstrating effectiveness” (p. 4 in [57]). Yet, as mentioned in our results, most authors in our sample that address issues relating to evidence in public health interventions stress that scientific evidence alone cannot guide and determine health policy and interventions decisions. Beyond evidentiary considerations, ethical impacts or issues must be considered, including unintended consequences of policies, such as stigma, negative impact on autonomy and individual choice, and negative perceptions [38, 47, 92]. Without such considerations, a sound evaluation of efficacy as well as a right balance of risks and benefits cannot be achieved.

The boundaries between research and public health monitoring and evaluation may be indistinct, and as such, the extent to which such evidence-generating activities should undergo ethical review has been debated [39, 48]. Yet, irrespective of whether the monitoring and evaluation qualifies as research, there is a definite need for ethical standards in practice. As mentioned by Gopichandran et al. [48], while there are several ethical frameworks in public health, none had focused on the monitoring and evaluation process. The framework proposed by these authors constitutes an ethical ground to guide ethical decision-making in the evaluation of public health interventions and can certainly be used for, and adapted to, the development of ethical guidelines for other specific nutrition-related public health interventions. In addition, all ethical frameworks mentioned in this article are valuable tools to develop ethical guidelines for the evaluation of nutrition-related public health interventions. The recommendations that are provided by these authors for the design and implementation process of policies are relevant when it comes to assessing their actual impact. Some of these frameworks [12] explicitly include post-implementation evaluation as an ethical requirement.

### Limitations

Beyond the limitations described in Section “Material and Methods: Step 2” and in the legends of figures and tables, we acknowledge that the use of different databases could provide records of publications that were not identified in this scoping review. Despite this limitation, we think that our sample captures the field of ethics when explicitly addressed in the literature related to nutrition-related public health interventions, but also illustrates the difficulties to practically cover such a broad field in a scoping review, and thus a priori in a systematic review. The presence in our final sample of 30 articles in which the “ethic*” word is not used while they are actually addressing ethical issues demonstrates the challenge in identifying relevant literature in a scoping review that includes all potential nutrition-related interventions. However, the results reported above clarify a complex field by illustrating the extent, range and nature of the ethical issues discussed in nutrition-related public health interventions.

## Conclusion

The results of this review illustrate the various natures, types, and scopes of existing (or planned) public health nutrition-related interventions, the widely differing contexts in which they are implemented, and the array of ethical issues that may arise. Ethical issues can only be addressed by taking into consideration the complexities of each specific setting. As a consequence, while general ethical frameworks or recommendations that follow from such consideration are certainly useful to draw attention to these issues, they cannot be expected to provide policy makers, implementors and other public health personnel with sufficient practical ethical guidance on how to achieve such goals in complex settings and specific public health nutrition interventions.

This scoping review also illustrates the methodological challenges that must be faced when conducting such a review and constitutes a needed and useful step in the design and achievement of future research seeking to identify ethical issues that are raised by nutrition-related public health interventions and in the development of ethical frameworks for policy makers and health professionals. We suggest that, given the complexity and diverse natures of interventions and contexts in the field of public health nutrition, future reviews should focus solely on specific interventions, without limiting their search to articles or studies that explicitly address ethical issues. Every item in the sample of publications should then be reviewed and analyzed in order, first, to identify ethical issues, addressed or not and, second, potential gaps in existing recommendations and guidelines relating to that specific nutrition-related intervention.

## Supporting information

S1 Table Queries in PubMed. (DOCX)

S2 Table Final sample. (XLSX)
