# Correction: Ethical issues in the development and implementation of nutrition-related public health policies and interventions: A scoping review

**DOI:** 10.1371/journal.pone.0192356

**Published:** 2018-02-05

**Authors:** Thierry Hurlimann, Juan Pablo Peña-Rosas, Abha Saxena, Gerardo Zamora, Béatrice Godard

S1 Table is a duplicate of Table 1. Please view the correct S1 Table here.

## Supporting information

S1 Table Queries in PubMed. (DOCX)
