# Supplementary material for: Ethical issues in the development and implementation of nutrition-related public health policies and interventions: A scoping review
Source: PLoS One. 2017 Oct 26;12(10):e0186897. doi: 10.1371/journal.pone.0186897 (PMC5658098; doi:10.1371/journal.pone.0186897)
Supplement: S1 Table — (DOCX) [file pone.0186897.s001.docx]

**Table 1: Most addressed issues linked to ethics in the whole sample (n=169), % per field**

|  | **Public health ethics**  **(no focus on nutrition)** | **Obesity** | **Non-**  **communicable diseases** | **Food security** | **Under-nutrition** | **Sustainability** | **Breastfeeding** | **Food safety** | **Food fortification** | **Vitamin / mineral supplement** |
| --- | --- | --- | --- | --- | --- | --- | --- | --- | --- | --- |
| Number of articles | (n=29) | (n=62) | (n=52) | (n=38) | (n=28) | (n=24) | (n=17) | (n=9)* | (n=6)* | (n=6)* |
| **Justice and equity** | 86.2 | 37.1 | 28.8 | 52.6 | 46.4 | 54.2 | 23.5 | 33.3 | 33.3 | 16.6 |
| **Beneficence, benefits** | 75.9 | 58.1 | 75 | 55.3 | 39.3 | 54.2 | 58.8 | 55.5 | 50 | 33.3 |
| **Non-maleficence, harms, risks** | 75.9 | 62.9 | 63.5 | 50 | 35.7 | 50 | 52.9 | 44.4 | 50 | 33.3 |
| **Accountability** | 65.5 | 46.8 | 30.8 | 44.7 | 50 | 58.3 | 29.4 | 44.4 | 50 | 16.6 |
| **Individual/parental autonomy** | 48.3 | 43.5 | 34.6 | 28.9 | 21.4 | 16.7 | 41.2 | 11.1 | 33.3 | - |
| **Stigmatization / discrimination** | 41.4 | 40.3 | 13.5 | 21 | 10.7 | 12.5 | 11.8 | - | - | - |
| **Partnerships – PPPs**** | 41.4 | 40.3 | 25 | 55.3 | 46.4 | 62.5 | 41.2 | 33.3 | 33.3 | 16.6 |
| **Community involvement – Participatory process** | 31 | 12.9 | 7.7 | 28.9 | 21.4 | 29.2 | 11.8 | 22.2 | - | - |
| **Social determinants of health** | 37.9 | 48.4 | 38.8 | 63.1 | 50 | 50 | 23.5 | 22.2 | 16.6 | - |
| **Individual or parental responsibility** | 27.6 | 46.8 | 21.1 | 31.6 | 17.8 | 20.8 | 23.5 | - | 16.6 | 16.6 |
| **Cultural factors and issues** | 27.6 | 37.1 | 40.4 | 50 | 39.3 | 58.3 | 41.2 | 33.3 | 16.6 | 16.6 |
| **Empowerment** | 24.1 | 11.3 | 19.2 | 15.8 | 7.1 | 8.3 | 11.8 | 11.1 | - | - |
| **Paternalism** | 24.1 | 22.6 | 11.5 | 7.9 | 3.6 | 4.2 | 23.5 | - | 33.3 | - |
| **Human rights** | 20.7 | 16.1 | 15.4 | 34.2 | 35.7 | 29.2 | 11.8 | 22.2 | 16.6 | 33.3 |
| **Conflict of interests** | 13.8 | 16.1 | 15.4 | 5.3 | 10.7 | 16.7 | 23.5 | 22.2 | 33.3 | 16.6 |
| **Stakeholders’ perceptions** | 6.9 | 9.7 | 15.4 | 5.3 | 10.7 | 8.3 | 23.5 | - | 33.3 | 16.6 |

*Percentages in this column must be taken with caution given the small sample. Articles may appear in more than one category. For instance, all articles in the category “stigmatisation” also appear in the category “Non-maleficence, harms, risks”. Articles that could not be accessed (n=8) could not be fully reviewed, thus they only appear in the categories that could be identified in title and abstract. The former fields “Health claims, food marketing, labeling & advertisement”, “Cash transfers, food stamps and other incentives” and “Food taxes and food bans” (see Fig 2) are now included as subcategories in other relevant fields (obesity, non-communicable diseases, etc.) as they constitute specific examples of public health interventions in these main fields. ** Public-private partnerships
