# Supplementary material for: Correction: Ethical issues in the development and implementation of nutrition-related public health policies and interventions: A scoping review
Source: PLoS One. 2018 Feb 5;13(2):e0192356. doi: 10.1371/journal.pone.0192356 (PMC5798837; doi:10.1371/journal.pone.0192356)
Supplement: S1 Table — (DOCX) [file pone.0192356.s001.docx]

| **Queries in PubMed** |
| --- |
| ((ethical[Title]) AND "public health"[Title/Abstract]) AND activit*[Title/Abstract]) |
| ((ethical[Title]) AND "public health"[Title/Abstract]) AND evidence[Title/Abstract]) |
| ((ethical[Title]) AND "public health"[Title/Abstract]) AND intervention[Title/Abstract]) |
| ((ethical[Title]) AND "public health"[Title/Abstract]) AND program*[Title/Abstract]) |
| ((ethical[Title]) AND "public health"[Title/Abstract]) AND guid*[Title/Abstract]) |
| ((ethical[Title]) AND "public health"[Title/Abstract]) AND polic*[Title/Abstract]) |
| ((ethical[Title]) AND "public health"[Title/Abstract]) AND recommend*[Title/Abstract]) |
| ((ethical[Title/Abstract]) AND "public health"[Title/Abstract]) AND recommend*[Title/Abstract]) |
| ((ethical[Title/Abstract]) AND "global health"[Title/Abstract]) AND recommend*[Title/Abstract]) |
| ((ethical[Title/Abstract]) AND "global health"[Title/Abstract]) AND intervention[Title/Abstract]) |
| ((ethical[Title/Abstract]) AND "global health"[Title/Abstract]) AND program*[Title/Abstract]) |
| ((ethic*[Title/Abstract]) AND "global health"[Title/Abstract]) AND nutrition[Title/Abstract]) |
| ((ethical[Title/Abstract]) AND "global health"[Title/Abstract]) AND nutrition[Title/Abstract]) |
| ((ethical[Title/Abstract]) AND "global health"[Title/Abstract]) |
| ((ethical[Title/Abstract]) AND undernutrition[Title/Abstract]) |
| ((ethical[Title/Abstract]) AND malnutrition[Title/Abstract]) |
| ((ethical[Title/Abstract]) AND malnutrition[Title/Abstract]) AND act*[Title/Abstract] |
| ((ethical[Title/Abstract]) AND undernutrition[Title/Abstract]) AND act*[Title/Abstract] |
| ((ethical[Title/Abstract]) AND undernutrition[Title/Abstract]) AND act*[Title/Abstract] |
| ((ethical[Title/Abstract]) AND undernutrition[Title/Abstract]) AND intervention[Title/Abstract] |
| ((ethical[Title/Abstract]) AND undernutrition[Title/Abstract]) AND intervention[Title/Abstract] |
| ((ethical[Title/Abstract]) AND malnutrition[Title/Abstract]) AND intervention[Title/Abstract] |
| ((ethical[Title/Abstract]) AND malnutrition[Title/Abstract]) AND program*[Title/Abstract] |
| ((ethical[Title/Abstract]) AND undernutrition[Title/Abstract]) AND program*[Title/Abstract] |
| ((ethical[Title/Abstract]) AND undernutrition[Title/Abstract]) AND evidence[Title/Abstract] |
| ((ethical[Title/Abstract]) AND malnutrition[Title/Abstract]) AND evidence[Title/Abstract] |
| ((ethical[Title/Abstract]) AND malnutrition[Title/Abstract]) AND "public health"[Title/Abstract] |
| ((ethical[Title/Abstract]) AND undernutrition[Title/Abstract]) AND "public health"[Title/Abstract] |
| ((ethical[Title/Abstract]) AND undernutrition[Title/Abstract]) AND recommend*[Title/Abstract] |
| ((ethical[Title/Abstract]) AND undernutrition[Title/Abstract]) AND polic*[Title/Abstract] |
| ((ethical[Title/Abstract]) AND undernutrition[Title/Abstract]) AND guid*[Title/Abstract] |
| ((ethical[Title/Abstract]) AND malnutrition[Title/Abstract]) AND guid*[Title/Abstract] |
| ((ethical[Title/Abstract]) AND malnutrition[Title/Abstract]) AND polic*[Title/Abstract] |
| ((ethical[Title/Abstract]) AND malnutrition[Title/Abstract]) AND recommend*[Title/Abstract] |
| ((ethical[Title/Abstract]) AND malnutrition[Title/Abstract]) AND recommend*[Title/Abstract] |
| ((ethical[Title/Abstract]) AND malnutrtion[Title/Abstract]) AND recommend*[Title/Abstract] |
| ((ethical[Title/Abstract]) AND nutri*[Title/Abstract]) AND recommend*[Title/Abstract] |
| ((ethical[Title/Abstract]) AND nutri*[Title/Abstract]) AND guid*[Title/Abstract] |
| ((ethical[Title/Abstract]) AND nutri*[Title/Abstract]) AND evidence[Title/Abstract] |
| ((ethical[Title/Abstract]) AND nutri*[Title/Abstract]) AND polic*[Title/Abstract] |
| ((ethical[Title/Abstract]) AND nutri*[Title/Abstract]) AND health[Title/Abstract] |
| ((ethic*[Title/Abstract]) AND nutri*[Title/Abstract]) AND health[Title/Abstract] |
| ((ethic*[Title/Abstract]) AND nutri*[Title/Abstract]) AND evidence[Title/Abstract] |
| ((ethic*[Title/Abstract]) AND nutri*[Title/Abstract]) AND "public health"[Title/Abstract] |
| ((ethic*[Title/Abstract]) AND nutri*[Title/Abstract]) AND activit*[Title/Abstract] |
| ((ethic*[Title/Abstract]) AND nutrition[Title/Abstract]) AND activit*[Title/Abstract] |
| ((ethic*[Title/Abstract]) AND nutrition[Title/Abstract]) AND evidence[Title/Abstract] |
| ((ethic*[Title/Abstract]) AND nutrition[Title/Abstract]) AND recommend*[Title/Abstract] |
| ((ethic*[Title/Abstract]) AND nutrition[Title/Abstract]) AND program*[Title/Abstract] |
| ((ethic*[Title/Abstract]) AND nutrition[Title/Abstract]) AND intervention[Title/Abstract] |
| ((ethic*[Title/Abstract]) AND nutrition[Title/Abstract]) AND "public health"[Title/Abstract] |
| ethic* AND nutrition AND polic* |
| ethic* AND nutrition AND public health |
